# Effects of Air Pollutants on Development of Allergic Immune Responses in the Respiratory Tract

**DOI:** 10.1080/10446670310001626535

**Published:** 2003

**Authors:** Laurel J. Gershwin

**Affiliations:** School of Veterinary Medicine Department of Pathology, Microbiology and Immunology University of California at Davis 2019 Haring Hall Davis CA 95616 USA

## Abstract

The increased incidence of allergic asthma in the human population worldwide has stimulated many explanatory theories. A concomitant decrease in air quality leads to epidemiological and laboratory-based studies to demonstrate a link between air pollutants and asthma. Specifically, ozone, environmental tobacco smoke, and diesel exhaust are associated with enhancement of respiratory allergy to inhaled allergens. This review summarizes the state of the knowledge, both human epidemiology and laboratory animal experiments, linking air pollution to allergy. Critical issues involve development of the lung and the fetal immune response, and the potential for substances like ozone and ETS in the air to modulate early immune responses with lifelong consequences.


*Clinical & Developmental Immunology*, June–December 2003 Vol. 10 (2–4), pp. 119–126

Taylor & Francis healthsciences

### Effects of Air Pollutants on Development of Allergic Immune Responses in the Respiratory Tract

LAUREL J. GERSHWIN*

*School of Veterinary Medicine, Department of Pathology, Microbiology and Immunology, University of California at Davis, 2019 Haring Hall, Davis, CA 95616, USA*

The increased incidence of allergic asthma in the human population worldwide has stimulated many explanatory theories. A concomitant decrease in air quality leads to epidemiological and laboratory-based studies to demonstrate a link between air pollutants and asthma. Specifically, ozone, environmental tobacco smoke, and diesel exhaust are associated with enhancement of respiratory allergy to inhaled allergens. This review summarizes the state of the knowledge, both human epidemiology and laboratory animal experiments, linking air pollution to allergy. Critical issues involve development of the lung and the fetal immune response, and the potential for substances like ozone and ETS in the air to modulate early immune responses with lifelong consequences.

*Keywords*: Air pollution; Asthma; Developmental immunology; IgE

### INTRODUCTION

The prevalence of allergic diseases, such as rhinitis ("hay fever") and asthma, has increased markedly during the past fifty years (Nicolai, 2002). This is a worldwide phenomenon as indicated by increased "hay fever" incidence in Japan from 3.8% in 1974 to over 10% currently (Salvi, 2001). The reported incidence of allergic rhinitis in the UK is reported to be 24%. Some references put the percentage of allergic individuals as high as 30% in Western countries. In fact, approximately 10% of schoolchildren are asthmatic, while in the 1950s only about 1% of children were asthmatic (Salvi, 2001). There is much evidence suggesting that the increase in allergic respiratory diseases, such as asthma and rhinitis is related to increased atmospheric concentrations of air pollutants, such as ozone (D'Amaato *et al.*, 2002), environmental tobacco smoke, and diesel exhaust particles.

It is well recognized that type 1 hypersensitivity diseases, such as rhinitis and asthma are initiated by exposure to antigens in the environment called allergens. These allergens are generally plant pollens, dust mites, and molds — substances that are difficult to avoid. Animal danders comprise a significant source of allergens in many allergic patients. The patient with rhinitis ("hay fever") has clinical signs that are related to the nasal cavity, with sneezing and conjunctivitis common sequelae. Asthma is a lung disease characterized by increased bronchial hyperreactivity, inflammation, and in the chronic state, airways remodeling. Most forms of asthma are found in individuals that are said to be "atopic", meaning that they have a genetic propensity to produce IgE antibodies against inhaled allergens.

Allergic diseases are characterized by the development of IgE antibodies that react with the allergens. These IgE antibodies bind firmly to high affinity IgE Fc receptors on mast cells in the tissues and upon exposure to the allergen that induced the IgE synthesis, the mast cells release mediators from their granules. The mediators (histamine, bradykinin, eosinophil chemotactic factors, etc.) cause the resultant clinical signs, such as lacrimation, nasal discharge, itching, and sneezing. In the asthmatic patient, additional effects include bronchial smooth muscle contraction, leading to a decrease in expiratory volume. Arachidonic acid cascades that are stimulated by allergen interaction with IgE on mast cells result in production of leukotrienes and prostaglandins, which have longer lasting smooth muscle contraction and chemotactic properties. The stimulus for IgE production evokes a response from T lymphocytes, called a T helper type 2 (Th2) response. This means that certain cytokines are produced that have an effect on B lymphocytes with resultant stimulation of IgE production. The presence of a Th2 cytokine profile is considered to be indicative of an allergic response (Gould *et al.*, 2003).

While the initial stimulus for the asthmatic response is IgE-mediated, chronic exposure to inhaled allergens generates a cellular influx and remodeling of the bronchial

*Corresponding author. Tel.: +1-530-752-6643. Fax: +1-530-752-4669.

ISSN 1740-2522 print/ISSN 1740-2530 online © 2003 Taylor & Francis Ltd
DOI: 10.1080/10446670310001626535

smooth muscle, with increased mucus cell production. A common method to evaluate the presence of IgE antibodies (and hence allergic/atopic sensitization to allergens) is the skin prick or intradermal test. In this test, a small amount of each potential allergen is injected into the skin. If the area develops a wheal within a few minutes, the presence of IgE-mediated mast cell degranulation is confirmed. This is a useful procedure to determine to which allergens (if any) an individual is allergic. It does not, however, relate directly to the presence of asthma. Asthma is defined as having airway constriction in response to inhalation of allergen. In atopic asthma, it is usual to have one or more positive skin test. (Gould *et al.*, 2003).

As stated above in recent years the incidence of allergic/atopic asthma has been increasing dramatically in industrialized countries. In fact, the number of asthma cases has more than doubled since 1998, resulting in an estimated 5,500 deaths yearly (Redd, 2002). This represents a major health problem in the United States. A variety of causes have been implicated for this increase, including: increases in vehicular exhaust, increased levels of air pollutants (ozone, nitrogen dioxide, and particulate matter), exposure to tobacco smoke, respiratory viral infection, and the recently coined "hygiene hypothesis". This hypothesis attributes the increase in asthma to movement of populations away from the traditional farm environment where exposure to microbes modulates the immune response away from the allergic phenotype (Matricardi and Ronchetti, 2001). While the hygiene hypothesis may provide insight into the immunological control of allergic responses in the lung, it is clearly not the single most important factor causing the "asthma epidemic". One has only to review the historical epidemiological reports that link asthma with air pollution to appreciate the impact of air quality on this disease (Folinsbee, 1993; Koren, 1995). This report focuses on the data that has been published linking air pollution and respiratory allergy, with particular emphasis on ozone.

### EPIDEMIOLOGICAL STUDIES

### Historical Studies

The association between increased incidence of asthma and air pollution is not new. In 1948 in Japan a syndrome called "Tokyo–Yokohama Asthma" was described. This syndrome occurred between September and May 1948 in previously normal adults who had recently moved to this highly industrialized area. The disease, characterized by a chronic nocturnal cough, wheezing, and shortness of breath, was rapidly progressive while the patient resided in the area and showed improvement when the patient left the area (Smith *et al.*, 1964). A group of patients composed primarily of U.S. military personnel and their dependents were studied. In all there were 426 reported cases, 28% of which had shown some previous allergic symptoms. The air stability had an effect on the syndrome, with disease exacerbation following days of stable air. Indeed in Tokyo the $SO_2$ often measured 0.072 ppm and the smog layer frequently reached up to 1,000 feet. In another study, Oshima *et al.* (1964) examined the indigenous population in the T–Y area and compared these individuals to those in Niigata area, where air pollution is much less. In that study, it was concluded that the workers in the T–Y area had increased incidence of respiratory disease. Moreover, the increase in disease among cigarette smokers and patients with a history of allergic disease hinted that air pollution might not only stimulate new allergic disease, but exacerbate conditions that were already present.

Another early study in Japan focused on school children from three severely polluted cities and compared these with one unpolluted city (Saku city) (Yoshida *et al.*, 1974). The sources of pollution included pulp and paper mills (Fugi city), electronic power plants, petroleum refineries (Chiba prefecture), and urban transportation (Tokyo). The presence of allergic disease was established using peripheral eosinophil counts, intradermal skin tests, and serum IgE levels. Increases and/or positive responses in all of these parameters are indicative of an allergic response. Results showed that Fuji city had a prevalence rate of asthma that was 2.19% compared with 0.94% in Saku city. Similarly, the prevalence rate for Tokyo was 2.74%. These data are from the early 1970s.

Other early epidemiological studies include a Nashville study which sought to correlate the frequency and severity of asthma attacks with the level of air pollution in Nashville, Tennessee. This 1961 study showed that there was a direct correlation between the $SO_4$ concentration in the air and the asthma attack rate (Zeidberg *et al.*, 1961).

For over forty years the increase in the ozone concentrations in the Los Angeles area has been followed and associations between high ozone levels and acute episodes of respiratory distress, including asthma have been documented. Indeed, hospital admissions were correlated with ozone levels in a 1966 report by Sterling *et al.* (1966). Another study focused on 157 asthma patients and had each of them keep a diary of their asthma. Air pollution data was supplied by the L.A. County Air Pollution Control District. Indeed, on days when oxidant values were high, a significant number of patients reported asthma attacks.

### Recent Studies

To study the relationship between the prevalence of atopy and photochemical air pollutants, a cross-sectional epidemiological survey was performed in 2604 primary school children living in seven communities in France. The gaseous air pollutants ($SO_2$, $NO_2$, and $O_3$) were measured during a two-month period. Skin prick tests were performed on the children and used to evaluate atopy. It should be noted that this study did not evaluate the presence of asthma or related pulmonary symptoms, but rather focused solely on skin sensitization. The results did not demonstrate any association between atopy and mean levels of the three air pollutants (Charpin *et al.*, 1999).

In another study of children exposed to different levels of air pollution in Italy, not only skin prick tests for atopy, but also respiratory function tests were performed. Comparing Milan, with high levels of air pollution (107 children), with Erba, a small rural town with low air pollution (113 children) no link between reduced lung function or the presence of atopy was demonstrated (Centanni *et al.*, 2001).

### Experimental Exposure Studies in Humans

Several studies have purposefully exposed human volunteers to various levels of ozone and other pollutants ($NO_2$ and $SO_2$) and then challenged with inhaled allergen while monitoring pulmonary function. Several studies have demonstrated an increased airway response to inhaled allergen following exposure to pollutants. For example, Molfino *et al.* (1991) showed that exposure to 120 ppb ozone for 1 h increased the bronchial sensitivity of asthmatics to ragweed challenge, as demonstrated by a decrease in the amount of allergen needed to cause a defined reduction in the forced expiratory volume ($FEV_1$). Other studies have shown similar effects: 250 ppb for 3 h (Jorres *et al.*, 1996). In contrast to these studies, Ball *et al.* (1996) demonstrated that pre-exposure to $O_3$ did not alter the amount of allergen necessary to induce a 15% reduction in the forced expiratory volume ($FEV_1$).

Jenkins *et al.* (1999) examined the pollutant dose effect of exposure to a combination of pollutants. Thus, eleven nonsmoking mildly asthmatic patients were involved in two protocols. In the first protocol the patients were exposed for 6 h to air, 100 ppb $O_3$, 200 ppb $NO_2$, and the combination of 100 ppb $O_2$ and 200 ppb $NO_2$. After exposure, the patients were challenged via aerosol with increasing doses of *D. pteronyssinus* (house dust mite) allergen. In the second protocol, the exposures to pollutants were only 3 h in duration. The concentrations of pollutants were greater (200 ppb $O_2$, 400 ppb $NO_2$, and 200 ppb $O_3$ and 400 ppb $NO_2$). These experiments demonstrated that exposure of mild atopic asthmatics for 3 h to 200 ppb $O_3$ and 400 ppb $NO_2$ alone or in combination resulted in a significant increase in lung sensitivity to inhaled allergen, although the effects were not additive. Moreover, the exposure for a shorter time to a higher concentration of pollutant was more effective in increasing the airway responsiveness to allergen challenge.

Accumulation of eosinophils in airway mucosa and/or lavage fluid is considered to be a hallmark of allergic disease. Thus, to determine the effect of 0.16 ppm ozone on allergic airway inflammation, Peden *et al.* (1997) exposed eight asthmatic patients with house dust mite sensitivity to ozone or clean air (with a 4 week washout period) and performed bronchoscopy 18 h later. Results showed that the ozone exposure was associated with increased eosinophilic inflammation in the lower airways of these patients, as compared with clear air (Peden, 1997).

In another study, Hiltermann *et al.* (1999) compared induced sputum with bronchoalveolar lavage (BAL) as sampling methods for evaluation of the cellular and cytokine response to ozone in asthmatics. There was a high correlation between eosinophil counts in the sputum and in the BAL fluid. In both samples the levels of eosinophils were elevated after exposure to 0.4 ppm ozone for 2 h. In addition, IL-8 and ECP were elevated and correlated between the two samples.

The synergy of ozone and allergen in promoting the duration of the asthmatic response was demonstrated by Vagaggini *et al.* (2002) in a study that utilized 12 subjects with mild persistent untreated asthma. After initial allergen challenge tests demonstrated an early and a delayed response to allergen challenge, these patients were exposed to allergen aerosol and then 24 h after allergen exposure they were exposed to ozone or air for 2 h. Pulmonary function tests were performed before and after these air or pollutant exposures. Sputum was examined for IL-8 and for cell numbers and types. The results showed that the exposure to ozone potentiated the eosinophilia, but did not alter the production of IL-8.

A study was performed in human subjects to evaluate and compare the allergic response after either single (125 or 250 ppb) or multiple exposures to ozone (125 ppb) or filtered air as control followed by allergen exposure the next day. In this study, mast cell tryptase and histamine, eosinophil enumeration in sputum, and exhaled nitric oxide were evaluated. The subjects were comprised of 22 with rhinitis and 11 with mild asthma. The mean concentration of methacholine required to produce a 20% fall in $FEV_1$ was significantly greater in the rhinitis group and the nitric oxide concentration during expiration was significantly lower in the rhinitis group than in the asthma group. Subjects in both groups had varying levels of IgE, with no statistical difference between groups. Results of this study were complex and not all of the parameters reached statistical significance. However, compared with filtered air the four times exposure to 125 ppb ozone was associated with higher total cell counts in sputum, with neutrophils and eosinophils comprising a greater percentage of cells in both rhinitis and asthma patients. The early phase FEV1 response to allergen challenge showed that subjects with rhinitis showed a significant decrease in this parameter after 250 ppb ($p = 0.002$) and four times exposure to 125 ppb ozone ($p = 0.04$). The subjects with asthma did not have a statistically significant change in this parameter. The authors compare the four times exposure with that amount of ozone that would be likely to be encountered during a week-long summer bike tour, stating that this exposure would likely enhance allergen responsiveness (Holz *et al.*, 2002).

### *In Vitro* Studies using Human Tissues and Cells

To examine the mechanisms involved in pollutant enhancement of asthma studies have been performed using either tissues or cells from asthmatic and control subjects. For example, to evaluate the effect of ozone and nitrogen dioxide on the permeability of bronchial epithelial cells from asthmatics compared with non-asthmatic subjects, Bayram *et al.* (2002) exposed cultured bronchial epithelial cells from both types of subjects to either air or pollutant for a period of 6 h. Cell permeability to $^{14}$C-labeled bovine serum albumin (BSA) was measured. The results showed that 10–100 ppb of $O_3$ and 200–400 ppb of $NO_2$ induced epithelial permeability, which was significantly greater in asthmatics than in non-asthmatics. These results pose an interesting possibility that pollutants may indeed facilitate sensitization to inhaled allergens by increasing access to underlying dendritic cells required for antigen processing in the lung.

Isolated human airways were used to examine the interaction of airway sensitization and ozone exposure for induction of airway hyper-responsiveness. The human lung tissue came from cancer patients undergoing lung resection. The tissue was used to prepare sections of bronchi for *in vitro* testing. In this study, tissues were sensitized to house dust mite allergen by incubation in serum from allergic patients. Then, the bronchial rings were exposed to 1 ppm ozone for 20 or 40 min and the dose/response of antigen to produce airway contraction was measured and compared with tissues that had not been exposed to ozone. The results indicated that the exposure to ozone potentiated the contractile response of the human bronchus in response to antigen (Roux *et al.*, 1999). While *in vitro* studies such as this only involve a small part of the dynamic interactions that occur in asthma, this study demonstrates a synergy of specific antigen and non-specific irritant on human bronchial responsiveness.

While a number of studies have demonstrated that ozone is capable of enhancing the reactivity of asthmatic individuals to allergens that they have been previously sensitized to, there is little good data demonstrating that ozone exposure alone can induce allergic sensitization in human subjects (Forster and Kuehr, 2000). These studies are most readily accomplished using animal models.

### Experimental Models

The value of animal models for evaluation of the effects of ozone and other pollutants on allergic respiratory disease is that animals can be isolated and exposed to known components without concurrent environmental contaminants. In addition, specific sensitization with known characterized allergens can be employed. There are, in fact, two main effects of ozone on the allergic/asthmatic response. First, one can examine the role of ozone in facilitation of sensitization. Secondly, the effects of ozone on elicitation or worsening of the asthmatic response can be examined. While the latter has been studied with chosen patient populations, animal models are more appropriate for evaluation of the sensitization arm of the allergic response. A variety of models have been used, including the guinea pig, mouse, brown Norway rat, monkey, and dog.

The first studies on the influence of air pollutants on allergic lung diseases/asthma were performed in ovalbumin sensitized guinea pigs by Matsumura (1970a,b). At that time the guinea pig was considered to be the preferred animal model for the study of type 1 hypersensitivity, since they are very sensitive to allergen and display a similar shock organ response as humans. Matsumura studied the effects of ozone, nitrogen dioxide, and sulfur dioxide on experimentally induced respiratory allergy using this model. Animals were exposed to ozone at 1, 5, or 10 ppm, nitrogen dioxide at 20, 40, or 70 ppm, or sulfur dioxide at 20, 60, 180, or 330 ppm for 30 min. This was followed by inhalation of either BSA or ovalbumin for 45 min. The process was repeated five to seven times. After the 5th or 6th antigen aerosol anaphylactic symptoms were observed. Mortality in the 10 ppm ozone and antigen aerosol group was 33.3% compared with 0% in the ambient air control group. Skin tests with antigen showed that allergic sensitization had indeed been accomplished without parenteral sensitization. Results showed that ozone of 5 ppm and greater were able to enhance sensitization to inhaled antigen (Matsumura, 1970a,b).

To further investigate the mechanisms responsible for this phenomenon, Matsumura labeled ovalbumin with $^{131}$I and aerosolized it to guinea pigs after a 30 min exposure to 8 ppm of ozone. After measuring the levels of the labeled ovalbumin in the blood, Matsumura concluded that a single 8 ppm 30 min exposure to ozone was sufficient to facilitate absorption of egg albumin from guinea pig lungs (Matsumura, 1970a,b).

Twenty years later Sumitomo *et al.* (1990) built upon Matsumura's early work and showed that exposure to ozone at 1, 3, and 5 ppm decreased the threshold of ovalbumin from 0.5 to 0.02% for provocation of airway hyperresponsiveness. In this study the effect of ozone on previously sensitized guinea pigs was also to elicit enhanced airway hyper-responsiveness. Thus, they concluded that ozone can not only facilitate sensitization but also enhance provocation in this guinea pig model.

Vargas *et al.* (1994) used the guinea pig model to examine airway hyper-responsiveness induced by ozone, allergen, and dual exposure. Ozone exposures were for 1 h at 3 ppm. Response to histamine was evaluated (ED50 determination). In the sensitized guinea pigs the histamine ED50 significantly decreased after antigen challenge. This decrease was greater in animals exposed to ozone than in air control animals.

In another study using guinea pigs, the effect of long term ozone exposure on airway hyper-responsiveness was examined in sensitized and non-sensitized animals. In addition, another group received concurrent ozone and antigen exposure. Two levels of ozone were examined (0.1 and 0.3 ppm) and exposures were for 4 h per day for 4 days per week for 24 weeks. The animals that were not sensitized did not show any effect of ozone exposure.

However, ozone exposure did exacerbate airway hyperresponsiveness to both specific and non-specific provocation in sensitized animals. This effect lasted for 4 weeks after termination of the exposures. Moreover, antibody responses to the antigens used in sensitization were significantly correlated with increases in airway hyperresponsiveness (Schlesinger *et al.*, 2002).

The guinea pig model was also used to evaluate the effect of ozone on nasal allergy. Iijima *et al.* (2001) exposed guinea pigs to filtered air or to 0.4 ppm ozone for 5 weeks. Once a week during these exposures a 1% solution of ovalbumin was administered by the intranasal route. By the third week of the protocol animals exposed to ozone and ovalbumin showed an increased number of sneezes during a 20 min period after ovalbumin administration as compared to other groups. By week 5 these animals were sneezing at the average rate of 15 sneezes in 20 min. The quantity of nasal secretions was also elevated, significantly by the sixth and final ovalbumin administration. Histopathology showed infiltration of eosinophils into the nasal epithelium and subepithelium. In the subepithelium the ozone and ovalbumin group showed a significant increase in cells compared with the air and ovalbumin exposed animals. While ozone was shown to enhance the symptoms of nasal allergy in the guinea pig model, the IgE antibody response to ovalbumin did not show a significant difference in ozone versus air-exposed animals.

The mouse is currently the model of choice by most immunologists when examining the allergic response. However, in the mid to late 1970s the mouse had not yet achieved such status. Nonetheless, pioneer studies on the effects of ozone on the development of allergic responses to inhaled ovalbumin were examined by Osebold and Gershwin (Osebold *et al.*, 1980). To examine the effect of cyclic ozone exposure on allergic sensitization mice were exposed to 0.8 ppm ozone for three days in alternate weeks for a total of three exposures and for four days in the last exposure. On the day after each ozone exposure mice received a 1% aerosol of ovalbumin for 30 min, after which they remained in ambient air until the next ozone cycle. During the last aerosol exposure mice were observed for signs of atopic reactivity. One week after the fourth and final ovalbumin aerosol, mice were injected with ovalbumin by the IV route and were observed for development of systemic anaphylactic shock. Results indicated that exposure to ozone enhanced anaphylactic sensitization when compared with similarly sensitized mice housed only in ambient air. One hundred percent of ovalbumin/ozone exposed mice developed clinical anaphylaxis compared with 74% of ovalbumin/air exposed mice. A second experiment using 0.5 ppm ozone showed a difference of 85% for the ozone/ovalbumin group compared with 5% in the ovalbumin/air group (Osebold *et al.*, 1980; 1988). IgE producing cells were also examined in the lungs of mice from these and other experiments. Ozone was shown to significantly increase the number of IgE containing cells in lungs of mice exposed to the pollutant and ovalbumin aerosol. The difference in the number of IgE cells in ozone exposed mice compared with those in ambient air was highly significant ($p < 0.006$) in the 0.8 ppm experiment previously described (Gershwin *et al.*, 1981). Data from these experiments showed that ozone exposure can enhance aerosol sensitization to antigen.

Work by another group using a very different protocol showed that the IgE response to parenterally administered ovalbumin was suppressed when the mice had been exposed to from 1 to 4 weeks of 0.8 ppm ozone followed by a single ovalbumin aerosol (6 min) 1 week prior to the parenteral injection (Ozawa *et al.*, 1985). This protocol is very different from the aforementioned one and the use of a parenteral exposure makes it less relevant to "real life" exposure than others that demonstrated an enhancing effect of ozone on the IgE response to aerosol-delivered ovalbumin.

More recently others also studied the effect of ozone on the allergic response using a mouse model. The studies by Neuhaus-Steinmetz *et al.* (2000) used ozone exposures ranging from 180 to 500 ug/m$^3$, which is comparable to 0.09–0.25 ppm. Mice were exposed 4 h three times per week for 4 weeks. Some mice also received ovalbumin aerosol five times per week throughout. Control mice received the ovalbumin without the ozone. Skin tests were performed to evaluate allergic sensitization. Other markers of allergic sensitization studied included: total IgE, ovalbumin specific IgE, IgG1, and IgG2a, Th1 and Th 2 cytokines, and leukotrienes C4, D4, and E4 in bronchoalveolar lung lavage fluid. In BALB/c mice, genetically Th2 skewed responders, ozone induced a dose dependent eosinophil response in BALF. IL-4 and 5 were increased in BALF, whereas interferon gamma remained unchanged. In addition skin test sensitivity and ovalbumin specific IgG1levels in serum were elevated in ozone exposed mice. Leukotriene concentrations in BALF were significantly increased in dual treated mice. The low IgE responder mouse strain, C57BL/6 was also evaluated in this protocol. Mice treated with only ovalbumin showed the expected Th1 antibody profile, while treatment with ozone and antigen was associated with a shift towards a Th2. Thus, ozone appeared to modulate the immune response toward the allergic phenotype in this mouse model. This study is particularly important because it shows that the effect of ozone on the non-atopic population could potentially induce allergy in those that would not be expected (from genetic data) to develop an IgE response to environmental allergens.

Another recent study using C57/BL/6 (a low IgE responder strain) mice concluded that ozone exposure does not increase allergic sensitization but enhances antigen-induced airway inflammation (Depuydt *et al.*, 2002). In this study, mice (0.1 ppm for 4 h ozone exposed or air) are "immunized" by antigen-pulsed syngeneic dendritic cells and then challenged with ovalbumin 2 weeks later. Other groups were similarly allergen exposed without ozone but received ozone (or air for control group) during the ovalbumin challenge. There was an increased eosinophilia in the BAL of the latter (ozone challenge) groups, but not in the groups that received ozone concurrent with the dendritic cells. While this study facilitated examination of the challenge phase of the allergic response, it does not adequately address sensitization. Use of a non-allergic mouse strain fails to model the atopic human population. In addition, administration of dendritic cells pulsed with allergen is not relevant to "real life" exposure. Finally, neither IgE nor airways hyper-responsiveness were examined as indicators of allergic sensitization/ responsiveness.

Another rodent model for allergy has been the Brown Norway rat. These rats were used by Wagner *et al.* (2002) in a recent study that examined the effect of ozone exposure on allergic rhinitis, in an ovalbumin-induced model. The ozone-sensitized rats were exposed to 0.5 ppm ozone for 8 h/day for either one or three consecutive days. Following the ozone exposure rats received either ovalbumin or saline intranasally. Twenty four hours after this antigen instillation the rats were sacrificed and the nasal tissues were examined. Results indicated that rats receiving ozone and allergen showed the presence of mucus-containing cells in the epithelium and increased inflammation. This study shows that in non-asthmatic subjects, allergic rhinitis "hay fever" can also be exacerbated by inhalation of ozone.

Some ozone-related experiments have been performed using a canine model. The dog is often naturally sensitized to Ascaris antigen due to early infection with the roundworm. This provides a convenient model for aerosol challenge and elicitation of allergic signs. Using this model the effects of ozone on allergic reactivity have been examined by several groups. One study by Spannhake using Ascaris sensitive dogs showed that mast cell mediators (histamine and the arachidonic acid metabolite prostaglandin D2 were decreased in mast cells present in lung lavage taken 30 min after exposure of dogs to 5 min of 1 ppm ozone, compared to dogs breathing only ambient air. The BAL cells were challenged *in vitro* by exposure to Ascaris antigen or an ionophore (Spannhake, 1996). The arachidonic acid metabolites, leukotrienes and prostaglandins, are generally considered to be late phase reactants that exert their effect hours after the primary allergen contact. Thus, timing for measurement of these mediators is critical.

Ozone has been shown to increase airway hyper-responsiveness in dogs, even in the absence of allergen challenge. Janssen *et al.* (1991) demonstrated that production of prostaglandins, especially E2, are important in this mechanism. Li *et al.* (1992) demonstrated that dogs with ozone-induced airway hyperresponsiveness also had a neutrophil influx in BAL. Using a monoclonal antibody to block the leukocyte adhesion to endothelial cells, they showed that the reduction of neutrophils and eosinophils by this treatment did not correlate with a change in ozone-induced airway hyper-responsiveness. This study demonstrates that ozone has a similar effect as allergen on induction of an influx of inflammatory cells into the lung, but that these cells are not responsible for the increase in airway hyper-responsiveness caused by ozone exposure.

The primate has been used infrequently as a model for asthma. Yet, the monkey is an ideal model for these studies because both the lung morphology and the immune system are more like that of the human than in other animal models. Using a cynomolgus monkey model in 1986 Biagini showed that exposure to 1 ppm ozone enhanced development of allergy to inhaled platinum (Biagini *et al.*, 1986). In this study, airway response to allergen and methacholine challenge as well as skin test reactivity were found to be significantly affected in the combined allergen and ozone group. The importance of this study is that it is the first primate study to show both functional and immunological enhancement of allergic responsiveness to allergen by ozone.

More recently a Rhesus monkey model of asthma is being used to examine the influence of ozone exposure on development of both immunological and structural attributes of asthma (Schelegle *et al.*, 2001). The combined exposure to epizootic ozone and house dust mite allergen has been shown to alter lung development (Evans *et al.*, 2003) and to alter the immune response (Miller *et al.*, 2003). These studies are ongoing and are expected to provide important mechanistic information regarding the interplay of ozone and allergen in the developing infant. This model is particularly relevant because it uses an allergen that is most common in asthmatic human subjects (*Dermatophygoides farinae*, the house dust mite). These studies are also the first to demonstrate morphologic, functional, and immunological data supporting the enhancement effect of ozone on allergic asthma. These studies use an epizootic and cyclic ozone and allergen exposure that is intended to replicate natural conditions. Moreover, some of the studies by this group use infant monkeys that serve as a model for the developing human child.

### Interactions of Ozone with Other Air Pollutants

Ambient air contains a mixture of pollutants. It is therefore not unexpected that a combination of pollutants would have a synergistic effect. Only in the research environment would we expect to see humans or animals exposed to single pollutants. Thus, studies have been performed in humans and in animal models using multiple pollutants.

A body of work has now been published demonstrating that diesel exhaust particles enhance the allergic response (including IgE and Th2 cytokine production) in humans and in murine models (Diaz-Sanchez, 1997; Nel *et al.*, 1998). Additional studies have shown that ambient concentrations of ozone can increase the allergenic activity of diesel exhaust (Madden *et al.*, 2000).

Studies on human subjects showed that neither $NO_2$ nor $SO_2$ alone appear to "prime" an asthmatic response to allergen, but when inhaled together these gases can increase sensitivity to subsequently inhaled allergen (Peden, 1997).

Matsumura used his guinea pig model to show that, like ozone, $NO_2$ and $SO_2$ are also able to enhance allergic reactivity. His data showed that singly a 30 min exposure to either 5 ppm ozone, 70 ppm $NO_2$ and 330 ppm. $SO_2$ cause enhancement of anaphylactic sensitization to antigen presented by the respiratory route (Matsumura, 1970a,b).

Several studies have demonstrated that $SO_2$ alone can influence allergic disease. Using the guinea pig model Riedel *et al.* (1988) showed that exposure to lower levels of $SO_2$ (0.1–16.6 ppm) for up to 12 weeks increased the antibody levels in serum and BAL fluid and enhanced bronchoconstriction in response to antigen. In another study with guinea pigs, results indicated that repeated exposure to low levels of sulfur dioxide may enhance the development of asthma ovalbumin-induced asthmatic reactions in guinea pigs (Park *et al.*, 2001).

Gilmour showed that Brown Norway rats sensitized and aerosol exposed to house dust mite allergen and then exposed to 5 ppm $NO_2$ for 3 h after the allergen aerosol had enhanced levels of serum IgE and mucosal IgA when compared with air exposed control rats (Gilmour, 1995).

The importance of these studies is that they demonstrate the likelihood that enhancement of allergic reactivity, and thus the recent asthma "epidemic" may be due, at least in part, to the synergism of multiple air pollutants interacting with environmental allergens.

### Animal Studies Demonstrate that ETS Exposure Enhances Allergic Sensitization to Inhaled Allergen in Primed Mice

Balb/c mice were exposed to ETS or filtered air for 6 h per day for 5 days a week at levels approximating those found in the homes of heavy smokers. Exposures were followed with ovalbumin parenteral sensitization and aerosol. Mice exposed to ETS had significantly more (compared with filtered air, OA sensitized) IgE, Th2 cytokines (after re-stimulation), and peripheral eosinophilia (Seymour *et al.*, 1997). In another study, pregnant mice were held in either ETS or ambient air. After parturition, neonates were retained in the same environment. Following parenteral priming, all mice demonstrated an increase in IgE production, this was most noticeable in female mice (Seymour *et al.*, 2002). In another study, ETS exposure facilitated intranasal sensitization of Balb/c mice to antigens of *Aspergillus fumigatus*. Pulmonary function testing revealed a distinct increase in airways hyperresponsiveness in the ETS group, as compared with sensitized filtered air controls. *Eosinophilia* was notable in BAL cell pellets (Seymour *et al.*, 2003).

### CONCLUSIONS

Understanding the reasons for the increasing prevalence of asthma in the industrialized countries requires consideration of multiple factors. Among these are changes in population hygiene and the absence of many infectious diseases common to previous generations, the occurrence of viral respiratory infections (such as respiratory syncytial virus) at an early age, exposure to indoor pollutants (such as side stream tobacco smoke) and increased levels of oxidant air pollutants and diesel exhaust particles. The effect of these factors and their interaction with genetics of the human subjects involved are the most likely contributing factors to the increase in allergic/atopic asthma. Epidemiological studies and animal models have supplied varied and significant information that should be useful in validation of the causal effects of the "asthma epidemic".

The studies reviewed herein demonstrate a strong argument that environmental air pollution is involved in the increase in asthma in the human population. However, the mechanisms involved are less clear. For example, the role of ozone in sensitization to allergen versus its role in exacerbation of the disease remains somewhat ambiguous. Some studies in the mouse model have shown a role for ozone in sensitization to inhaled allergen. Exactly how ozone mediates this effect at the cellular and molecular level will require additional study. Similarly mouse studies on the effects of environmental tobacco smoke show clear stimulation of a Th2 cytokine response and resultant IgE production. The role of pre-natal and neonatal exposure have just begun to be investigated, but data obtained thus far suggests that in utero exposure to tobacco smoke modulates the already Th2 biased immune response towards a strong Th2 phenotype in the offspring and subsequent allergy.

### *References*

Ball, B.A., Folinsbee, L.J., Peden, D.B. and Kehrl, H.R. (1996) "Allergen bronchoprovocation of patients with mild allergic asthma after ozone exposure", *J. Allergy Clin. Immunol.* **98**, 563–572.

Bayram, H., Rusznak, C., Khair, O.A., Sapsford, R.J. and Abdelaziz, M.M. (2002) "Effect of ozone and nitrogen dioxide on the permeability of bronchial epithelial cell cultures of non-asthmatic and asthmatic subjects", *Clin. Exp. Allergy* **32**, 1285–1292.

Biagini, R.E., Moorman, W.J., T, R.L. and Bernstein, I.L. (1986) "Ozone enhancement of platinum asthma in a primate model", *Am. Rev. Respir. Dis.* **134**, 719–725.

Centanni, S., Di Marco, F., Castagna, F., Santus, P., Guarnieri, R. and Allegra, L. (2001) "Atopy prevalence and spirometric performance in asymptomatic schoolchildren exposed to air pollution", *Monaldi Arch. Chest Dis.* **56**, 304–308.

Charpin, D., Pascal, L., Birnbaum, J., Armengaud, A., Sambuc, R., Lanteaume, A. and Vervloet, D. (1999) "Gaseous air pollution and atopy", *Clin. Exp. Allergy* **29**, 1474–1480.

D'Amaato, G., Liccardi, G., D'Amato, M. and Caxxola, M. (2002) "Respiratory allergic diseases induced by outdoor air pollution in urban areas", *Monaldi Arch. Chest Dis.* **57**, 161–163.

Depuydt, P.O., Lambrecht, B.N., Joos, G.F. and Pauwels, R.A. (2002) "Effect of ozone exposure on allergic sensitization and airway inflammation induced by dendritic cells", *Clin. Exp. Allergy* **32**, 391–396.

Diaz-Sanchez, D. (1997) "The role of diesel exhaust particles and their associated polyaromatic hydrocarbons in the induction of allergic airway disease", *Allergy* **52**, 52–56, Discussion 57-58.

Evans, M.J., Fanucchi, M.V., Baker, G.L., van Winkle, L.S., Pantle, L.M., Nishio, S., Schelegle, E.S., Gerswhin, L.J., Miller, L.A., Hyde, D.H., Sannes, P.L. and Plopper, C.G. (2003) "Atypical development of the tracheal basement membrane zone of infant rhesus monkeys exposed to ozone and allergen", *Am. J. Physiol. Lung Cell. Mol. Physiol.*.
Folinsbee, L.J. (1993) "Human health effects of air pollution", *Environ. Health Perspect.* **100**, 45–56.
Forster, J. and Kuehr, J. (2000) "The role of ozone", *Pediatr. Allergy Immunol.*(Suppl. 13), 23–25.
Gershwin, L.J., Osebold, J.W. and Zee, Y.C. (1981) "ImmunoglobulinE-containing cells in mouse lung following allergen inhalation and ozone exposure", *Int. Arch. Allergy Appl. Immunol.* **65**, 266–277.
Gilmour, M.I. (1995) "Interaction of air pollutants and pulmonary allergic responses in experimental animals", *Toxicology* **105**, 335–342.
Gould, H.J., Sutton, B.J., Beavil, A.J., Beavil, R.L., McCloskey, N., Coker, H.A., Fear, D. and Smurthwaite, L. (2003) "The biology of IgE and the basis of allergic disease", *Annu. Rev. Immunol.* **21**, 579–628.
Hiltermann, J.T., Lapperre, T.S., Van Bree, L., Steerenberg, P.A., Brahim, J.J., Sont, J.K., Sterk, P.J., Hiemstra, P.S. and Stolk, J. (1999) "Ozone-induced inflammation assessed in sputum and bronchial lavage fluid from asthmatics", *Free Radic. Biol. Med.* **27**, 1448–1454.
Holz, O., Micke, M., Paasch, K., Timm, P., Richter, K., Magnussen, H. and Jorres, R.A. (2002) "Repeated ozone exposures enhance bronchial allergen responses in subjects with rhinitis or asthma", *Clin. Exp. Allergy* **32**, 681–689.
Iijima, M.K., Kobayashi, T., Kamada, H. and Shimojo, N. (2001) "Exposure to ozone aggravates nasal allergy-like symptoms in guinea pigs", *Toxicol. Lett.* **123**, 77–85.
Janssen, L.J., O'Byrne, P.M. and Daniel, E.E. (1991) "Mechanism underlying ozone-induced *in vitro* hyperresponsiveness in canine bronchi", *Am. J. Physiol.* **261**, L55–L62.
Jenkins, H.S., Devalia, J.L., Mister, R.L., Bevan, A.M., Rusznak, C. and Davies, R.J. (1999) "The effect of exposure to ozone and nitrogen dioxide on the airway response of atopic asthmatics to inhaled allergen: dose- and time-dependent effects", *Am. J. Respir. Crit. Care Med.* **160**, 33–39.
Jorres, R., Nowak, E. and Magnussen, H. (1996) "Effect of ozone exposure on allergen responsisveness in subjects with asthma or rhinitis", *Am. J. Resp. Crit. Care Med.* **153**, 56–64.
Koren, H.S. (1995) "Associations between criteria air pollutants and asthma", *Environ Health Perspect.* **103**, 235–242.
Li, Z., Daniel, E.E., Lane, C.G., Arnaout, M.A. and O'Byrne, P.M. (1992) "Effect of an anti-Mo1 MAB on ozone-induced airway inflammation and airway hyperresponsiveness in dogs", *Am. J. Physiol.* **263**, L723–L726.
Madden, M.C., Richards, J.H., Dailey, L.A., Hatch, G.E. and Ghio, A.J. (2000) "Effect of ozone on diesel exhaust particle toxicity in rat lung", *Toxicol. Appl. Pharmacol.* **168**, 140–148.
Matricardi, P.M. and Ronchetti, R. (2001) "Are infections protecting from atopy?", *Curr. Opin. Allergy Clin. Immunol.* **1**, 413–419.
Matsumura, T. (1970a) "The effects of ozone, nitrogen dioxide, and sulfur dioxide on the experimentally induced allergic respiratory disorder in guinea pigs. I. The effect on sensitization with albumin through the airway", *Am. Rev. Respir. Dis.* **102**, 430–437.
Matsumura, Y. (1970b) "The effects of ozone, nitrogen dioxide, and sulfur dioxide on the experimentally induced allergic respiratory disorder of guinea pigs. II. The effects of ozone on the absorption and the retention of antigen in the lung", *Am. Rev. Resp. Dis.* **102**, 438–443.
Miller, L.A., Hyde, D.M., Gershwin, L., Schelegle, E.S., Fanucchi, M.V., Evans, M.J., Gerriets, J.E., Putney, L.F., Stovall, M.Y., Tyler, N.K., Usachenko, J.L. and Plopper, C.G. (2003) "The effect of house dust mite aeroallergen and air pollutant exposures during infancy", *Chest* **123**, 434S.
Molfino, N.A., Wright, S.C., Katz, I., Tarlo, S., Silverman, F., McClean, P.A., Szalai, J.P., Raizenne, M., Slutsky, A. and Zamel, N. (1991) "Effect of low concentrations of ozone on inhaled allergen responses in asthmatic subjects", *Lancet* **338**, 199–203.
Nel, A.E., Diaz-Sanchez, D., Ng, D., Hiura, T. and Saxon, A. (1998) "Enhancement of allergic inflammation by the interaction between diesel exhaust particles and the immune system", *J. Allergy Clin. Immunol.* **102**, 539–554.
Neuhaus-Steinmetz, U., Uffhausen Herz, U. and Renz, H. (2000) "Priming of allergic immune responses by repeated ozone exposure in mice", *Am. J. Respir. Cell. Mol. Biol.* **23**(2), 228–233.
Nicolai, T. (2002) "Pollution, environmental factors and childhood respiratory allergic disease", *Toxicology* **181**, 317–321.
Osebold, J.W., Gershwin, L.J. and Zee, Y.C. (1980) "Studies on the enhancement of allergic lung sensitization by inhalation of ozone and sulfuric acid aerosol", *J. Environ. Pathol. Toxicol.* **3**, 221–234.
Osebold, J.W., Zee, Y.C. and Gershwin, L.J. (1988) "Enhancement of allergic lung sensitization in mice by ozone inhalation", *Proc. Soc. Exp. Biol. Med.* **188**, 259–264.
Oshima, Y., Ishizaki, T., Miyamoto, T., Shimizu, T., Ahis, R. and Kabe, J. (1964) "Air pollution and respiratory diseases in the Tokyo–Yokohama area", *Am. Rev. Respir. Dis.* **90**, 578–581.
Ozawa, M., Fujmaki, H., Imai, T., Honda, Y. and Watanabe, N. (1985) "Suppression of IgE antibody production after exposure to ozone in mice", *Int. Arch. Allergy Appl. Immunol.* **76**, 16–19.
Park, J.K., Kim, Y.K., Lee, S.R., Cho, S.H., Min, K.U. and Kim, Y.Y. (2001) "Repeated exposure to low levels of sulfur dioxide ($SO_2$) enhances the development of ovalbumin-induced asthmatic reactions in guinea pigs", *Ann. Allergy Asthma Immunol.* **86**, 62–67.
Peden, D.B. (1997) "Mechanisms of pollution-induced airway disease: *in vivo* studies", *Allergy* **52**, 37–44.
Peden, D.B., Boehlecke, B., Horstman, D. and Devlin, R. (1997) "Prolonged acute exposure to 0.16 ppm ozone induces eosinophilic airway inflammation in asthmatic subjects with allergies", *J. Allergy Clin. Immunol.* **100**, 802–808.
Redd, S.C. (2002) "Asthma in the United States: burden and current theories", *Environ. Health Perspect.* **110**, 557–560.
Riedel, F., Kramer, M., Scheibenbogen, C. and Rieger, C.H. (1988) "Effects of $SO_2$ exposure on allergic sensitization in the guinea pig", *J. Allergy Clin. Immunol.* **82**, 527–534.
Roux, E., Hyvelin, J.-M., Savineau, J.-P. and Marthan, R. (1999) "Human isolated airway contraction", *Am. J. Crit. Care Med.* **160**, 439–445.
Salvi, S. (2001) "Pollution and allergic airways disease", *Curr. Opin. Allergy Clin. Immunol.* **1**, 35–41.
Schelegle, E.S., Gershwin, L.J., Miller, L.A., Fanucchi, M.V., van Winkle, L.S., Gerriets, J.P., Walby, W.F., Omlor, A.M., Buckpitt, A.R., Tarkington, B.K., Wong, V.J., Joad, J.P., Pinkerton, K.B., Wu, R., Evans, M.J., Hyde, D.M. and Plopper, C.G. (2001) "Allergic asthma induced in rhesus monkeys by house dust mite (*Dermatophagoides farinae*)", *Am. J. Pathol.* **158**, 333–341.
Schlesinger, R.B., Cohen, M.D., Gordon, T., Nadziejko, C., Zelikoff, J.T., Sisco, M., Regal, J.F. and Menache, M.G. (2002) "Ozone differentially modulates airway responsiveness in atopic versus nonatopic guinea pigs", *Inhal. Toxicol.* **14**, 431–457.
Seymour, B.W., Pinkerton, K.E., Friebertshauser, K.E., Coffman, R.L. and Gershwin, L.J. (1997) "Second-hand smoke is an adjuvant for T helper-2 responses in a murine model of allergy", *J. Immunol.* **159**, 6169–6175.
Seymour, B.W., Friebertshauser, K.E., Peake, J.L., Pinkerton, K.E., Coffman, R.L. and Gershwin, L.J. (2002) "Gender differences in the allergic response of mice neonatally exposed to environmental tobacco smoke", *Dev. Immunol.* **9**, 47–54.
Smith, R.B.W., Kolb, E.J., Phelps, H.W., Weiss, H.A. and Hollinden, A.B. (1964) "Tokyo–Yokohama asthma. An area specific air pollution disease", *Arch. Environ. Health* **8**, 805–817.
Spannhake, E.W. (1996) "Down-regulation of canine airway mast cell function following exposure to ozone *in vivo*", *Exp. Lung Res.* **22**, 163–178.
Sterling, T.D., Phair, J.I., Pollack, S.V. and Schumsky, D.A. (1966) "Urban morbidity and air pollution", *Arch. Environ. Health* **13**, 158–170.
Sumitomo, M., Nishikawa, M., Fukuda, T., Kaneko, T., Suzuki, S. and Okubo, T. (1990) "Effects of ozone exposure on experimental asthma in guinea pigs sensitized with ovalbumin through the airway", *Int. Arch. Allergy Appl. Immunol.* **93**, 139–147.
Vagaggini, B., Taccola, M., Cianchetti, S., Carnevali, S., Bartoli, M.L., Bacci, E., Dente, F.L., di Franco, A., Giannini, D. and Paggiaro, P.L. (2002) "Ozone exposure increases eosinophilic airway response induced by previous allergen challenge", *Am. J. Respir. Crit. Care Med.* **166**, 1073–1077.
Vargas, M.H., Segura, P., Campos, M.G., Hong, E. and Montano, L.M. (1994) "Effect of ozone exposure on antigen-induced airway hyperresponsiveness in guinea pigs", *J. Toxicol. Environ. Health* **42**, 435–442.
Wagner, J.G., Hotchkiss, J.A. and Harkema, J.R. (2002) "Enhancement of nasal inflammatory and epithelial responses after ozone and allergen coexposure in Brown Norway rats", *Toxicol. Sci.* **67**, 284–294.
Yoshida, R., Motomiya, K., Saito, H. and Funabashi, S. (1974) Clinical Implications Air pollution Research (Publishing Sciences Group, Inc., Acton, MA), pp 165–182.
Zeidberg, L.D., Prindle, R.A. and Landau, E. (1961) "The Nashville air pollution study. I. Sulfur dioxide and baronchial asthma: a preliminary report", *Am. Rev. Respir. Dis.* **84**, 489–503.

